# Re-Audit of Use of Psychotropic Medication in Children and Adolescents Attending Barnet’s Service for Children and Adolescents With Neurodevelopmental Disorders (SCAN) Team

**DOI:** 10.1192/bjo.2025.10564

**Published:** 2025-06-20

**Authors:** Noemi Cortis, Janaki Bansal

**Affiliations:** North London Mental Health Partnership, London, United Kingdom

## Abstract

**Aims:** This re-audit assessed whether Barnet’s Service for Children and Adolescents with Neurodevelopmental disorders (SCAN) prescribing practices are in line with the National Institute for Care and Excellence (NICE) guidelines and the “Stopping Overuse of Medication in People with Learning Disability, Autism or both” (STOMP) and “Supporting Treatment and Appropriate Medication in Paediatrics” (STAMP) pledge. It also looked at whether psychotropic prescribing practices changed following the introduction of Positive Behavioural Support (PBS) Workshops in SCAN.

**Methods:** The sample consisted of 161 patients attending Barnet SCAN, Holly Oak Unit in Edgware Hospital as from January 2025. Electronic Patient Records via Rio were reviewed with data gathered on presence of LD and/or neurodevelopmental disorder, comorbid mental illness, documented use of therapeutic interventions and psychotropic medication prescribed.

**Results:** 88 out of 161 children and adolescents (55%) were on psychiatric medication. 48 of the children on psychotropic medication (55%) had a diagnosis of Attention Deficit Hyperactivity Disorder (ADHD), whilst 68 had a diagnosis of Autism Spectrum Disorder (ASD) (77%). 40 children (45%) had a diagnosis of both ADHD and ASD.

The number of clients offered therapeutic interventions increased from 50% to 91%. There was a higher number of young people prescribed psychotropics despite a rise in nonpharmacological interventions (19% in 2020 vs 24%).

**Conclusion:** The rise in use of psychotropic medication could be secondary to the increasing acuity and complexity of cases presenting to the SCAN team post COVID pandemic. The initial audit took place during lockdown, during which fewer cases were being seen by mental health services. COVID-19 had a profound negative impact on children’s mental health, behaviour, social skills and learning overall.

SCAN is working on pursuing further training in other therapeutic modalities including the ‘Intensive Interaction’ Course. SCAN will also continue promoting PBS through parenting programmes, individual sessions and psychoeducation.

